# The Effect of Adipose-Derived Stem Cells on Wound Healing: Comparison of Methods of Application

**DOI:** 10.1155/2019/2745640

**Published:** 2019-09-17

**Authors:** Hyeonwoo Kim, Mi Ri Hyun, Sang Wha Kim

**Affiliations:** Department of Plastic and Reconstructive Surgery, Seoul National University Hospital, Seoul, Republic of Korea

## Abstract

Impaired wound healing is a significant medical problem. Recently, cell-based therapy focused on stem cells has been developed to overcome the challenges of defective wound healing. In this study, we aimed to evaluate the effectiveness of adipose-derived stem cells (ASCs) in promoting wound healing, using different techniques for administering them. Dorsal full-thickness skin defects (1 × 1 cm) were created in three groups of mice that received intravenous ASCs by intravenous injection, intramuscular injection, and topical application, respectively. Three control groups received saline in the same ways. Wound healing was assessed clinically, wounds were examined histologically, and GFP-labelled ASCs were detected with an IVIS imaging system. The results revealed that ASCs accelerated wound healing independent of their mode of administration. Histological examination showed that the ASCs accelerated reepithelialization, and IVIS analysis indicated that many ASCs were present in the wound area and disappeared after wound healing.

## 1. Introduction

Impaired wound healing is a significant medical problem with a clinical and socioeconomic cost. Recent advances in regenerative medicine have revealed promising approaches to overcoming this probem [1, 2]. These are mostly focused on stem cells, which are specialized self-renewing cells with the ability to differentiate into multiple cell types [3].

Mesenchymal stem cells can be isolated from various sites, including bone marrow, adipose tissue, and cord blood [4–7]. Bone marrow-derived stem cells (BMSCs) can facilitate tissue repair by producing growth factors, cytokines, and extracellular matrix [6, 8] and promoting the migration of other cells [9]. Fathke et al. have shown that BMSCs are involved in reconstituting dermal fibroblast populations and healing wounds [8]. Adipose-derived stem cells (ASCs) are multipotent cells in adipose tissue with characteristics similar to those of BMSCs [4, 10]. However, in contrast to the invasive procedure needed to harvest BMSCs and their low yields, ASCs are easy to harvest and there is minimal donor morbidity. [5, 11] Since the first report by Zuk et al. [12, 13], many studies have confirmed that ASCs have the same favorable effects as BMSCs on wound repair, immunomodulation, and antiapoptotic activity [12–16].

In this study, we evaluated the effectiveness of ASCs in promoting wound healing in a rodent full-thickness dorsal excisional wound defect model, using various methods for administering the cell, namely intravenous, intramuscular, and topical application.

## 2. Methods

### 2.1. Isolation and Culture of Adipose-Derived Stem Cells

Healthy pure transgenic mice 3-5 weeks of age and expressing green fluorescent protein (GFP) were used to prepare the implanted ASCs. They were fed normally and were used after two weeks of adaptation.

Under anaesthesia, subcutaneous adipose tissue was harvested from the inguinal region of GFP-transgenic mice. About 1 mL of adipose tissue was harvested from inguinal fat pads and washed with PBS (Invitrogen, Carlsbad, CA, USA). It was minced with scissors into pieces of 1 mm^3^ and digested with 0.2% type I collagenase (Sigma-Aldrich, St. Louis, MO, USA) in a shaking incubator for 1 hour at 37°C. The resulting cell suspension was centrifuged at 1200 rpm for 10 min, the supernatant discarded, and the sediment used to prepare a cell suspension in Dulbecco's Modified Eagle's Medium (DMEM) containing 50 mL/L foetal bovine serum (Sigma-Aldrich, St. Louis, MO, USA). Samples of cells were plated in 100 mm^2^ tissue culture plates and maintained at 37°C in 5% carbon dioxide. The medium was changed after 24 h and every 3 days thereafter. The ASCs were harvested at 90% confluence and passaged at a 1 : 3 dilution.

Third-passage ASCs were used for all the experiments. Before application, ASCs were detached with 0.25% trypsin-EDTA and centrifuged at 250*g* at room temperature. The supernatant was discharged and the pellet was resuspended in the medium. Then, the cells were filtered through a 40 *μ*m cell strainer and diluted into a 1 × 10^6^/100 *μ*L single-cell suspension in EP tubes.

### 2.2. Immunofluorescence Staining

Passage 3 ASCs were seeded into 6-well culture plates at 2 × 10^5^ cells/well and cultured in DMEM-10% FBS for 3 days. The cells were washed with PBS, fixed with 4% paraformaldehyde for 30 min, and incubated with primary CD34, CD45, CD73, and CD105 antibodies (1 : 200, Abcam, Cambridge, UK) overnight. The cells were then washed three times with PBS and reacted with rabbit anti-mouse immunoglobulin G (IgG; 1 : 200, Abcam) for 45 min. Fluorescence images of the stained cells were obtained under an inverted fluorescence microscope (Leica-DMI4000B+DFC405C, Leica Microsystems, Wetzlar, Germany) and analysed with the Leica LAS X imaging editing program.

### 2.3. Wound Healing

All procedures were conducted in accordance with the guidelines of the Animal Welfare Committee of Seoul National University. Six-week-old male C57BL/7 mice (*n* = 36), weighing approximately 20 g, were used. They were maintained on an automatic 12 h light/dark cycle and were fed standard mouse food and water. All animals were checked daily for signs of inflammation, ulceration, and other side effects.

The surgery was performed under general anaesthesia in standard sterile conditions. A 1 × 1 cm full-thickness wound was created on the backs of 36 mice. The corners of the 1 × 1 cm square gaps were sutured to underlying muscle to prevent wound contraction. The mice were divided into 6 groups: three experimental groups received, respectively, intravenous injection of 1 mL of 1 × 10^6^ ASCs (ASCs/IV), intramuscular injection of 1 mL of 1 × 10^6^ ASCs (ASCs/IM), and topical application of 1 mL of 1 × 10^6^ ASCs mixed with fibrin gel (Tissucol Duo Quick, Baxter, Austria) (ASCs/fibrin). Three control groups received saline by the same routes (Saline/IV, Saline/IM, and Saline/fibrin).

### 2.4. Estimating Wound Healing Areas

After surgery on day 0, wound areas were measured. The sizes of the wounds were measured on days 1, 3(4), 7(9), and 14 after surgery. Digital photographs were taken and the wound area was determined using Image Analyser Software (ImageJ software version 2, National Institutes of Health, Bethesda, MD, USA).

### 2.5. Histological Examination

Mice were sacrificed on day 14. The wounded tissue and the surrounding skin were carefully excised, pinned, and fixed in 10% formalin overnight at 4°C. Sections were stained with hematoxylin and eosin to examine wound status.

### 2.6. Fluorescence Imaging

GFP-labelled ASCs were monitored using an IVIS Imaging System Series 200 (Caliper Life Science, Hopkinton, MA, USA) to investigate their engraftment and distribution over the wound area. GFP-labelled ASCs could to be detected with the IVIS imaging system.

### 2.7. Statistical Analysis

SPSS 20.0 Software (SPSS Inc., Chicago, IL, USA) was used for statistical analysis. Measurement data were expressed as means ± standard deviations. Comparisons between groups were performed with Student's *t*-test or one-way ANOVA. A value of *p* < 0.05 was considered statistically significant.

This study was approved by the Ethics Committee of the Seoul National University.

## 3. Results

### 3.1. Characterization of ASCs

**Figure 1:**
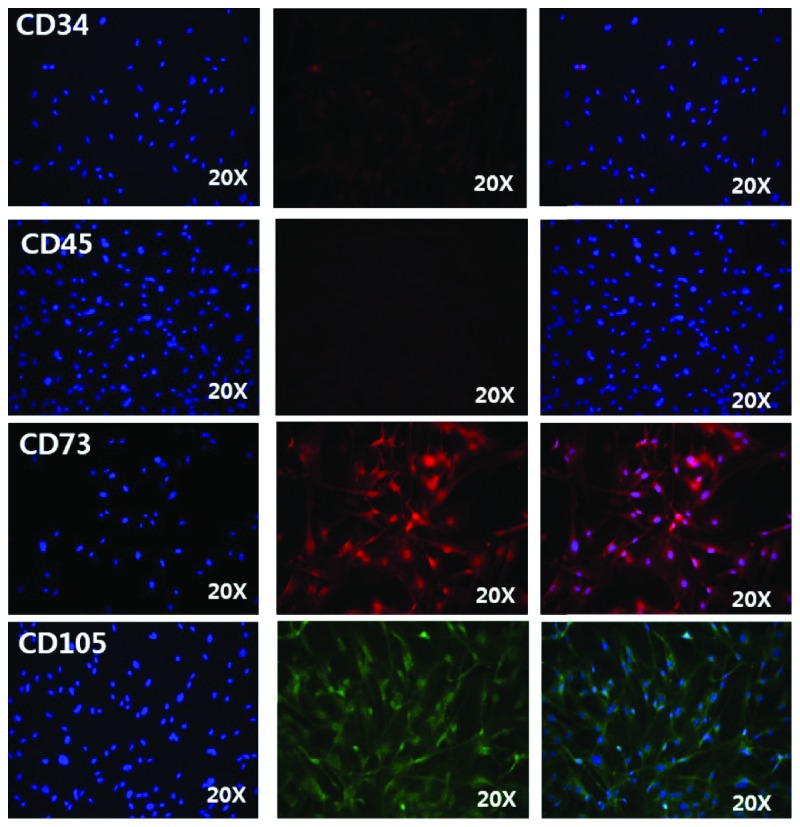
Characterization of ASCs. Immunofluorescence staining shows that the cultured ASCs were positive for cell markers CD73 and CD105 and negative for CD34 and CD45.

The ASCs expressed high levels of the stem cell markers CD73 and CD105 as judged by immunofluorescence staining. In contrast, they were negative for CD34 and CD45 (specific hematopoietic cell markers) (Figure 1).

### 3.2. Treatment with ASCs Accelerated Wound Healing

**Figure 2:**
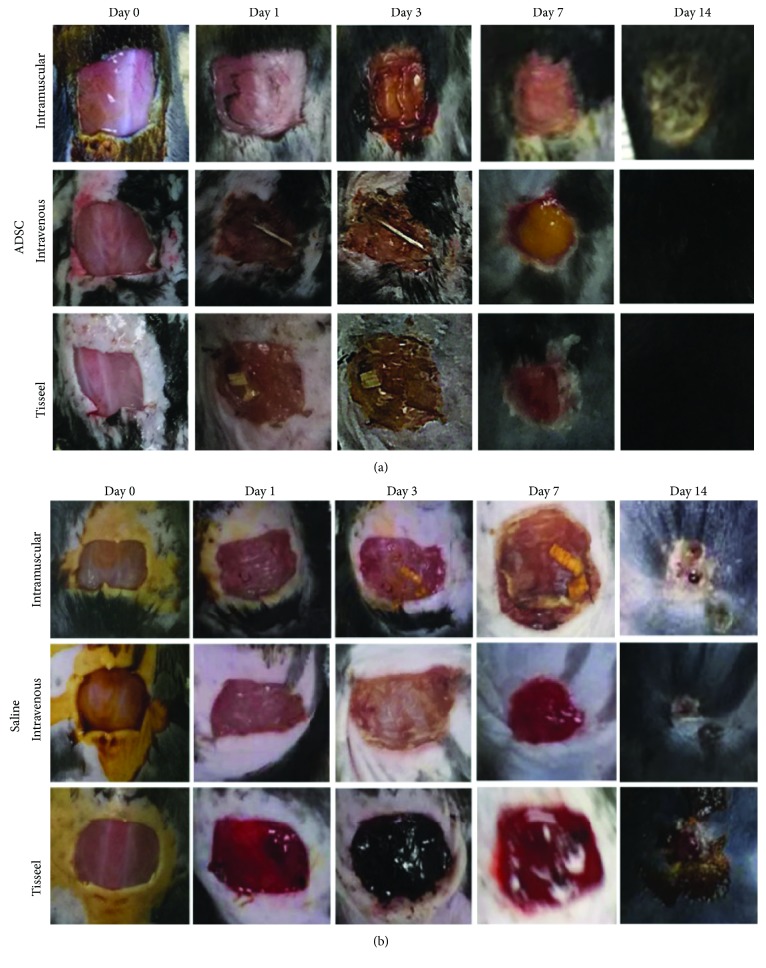
Effect of ASCs on the time course of wound closure. All wounds had an initial area of around 1 × 1 cm. (a) The representative gross appearance of the wound healing with different methods of applying ASCs on postoperative days 0, 1, 3, 7, and 14. No differences were observed between the different treatments on day 1, but from day 3 to day 7, an acceleration of wound healing was observed in all three treated groups. By day 14, there was 95% epithelization in all treated groups. (b) Gross appearance of the wound healing on postoperative days 0, 1, 3, 7, and 14 in the control groups receiving saline. The wound size was not reduced until day 7. By day 14, there was 60% epithelization in control groups.

Overall, the wound healing process was accelerated in the ASC-treated mice compared to the controls. All wounds had an initial area of around 1 cm^2^. Figures 2(a) and 2(b) show representative digital images of the evolution of wound healing in each group on days 0, 1, 3, 7, and 14 after surgery. No differences were observed between the different treatments on day 1, but from day 3 to day 7 a significant acceleration of wound healing was observed in all three treated groups compared to the controls.

**Figure 3:**
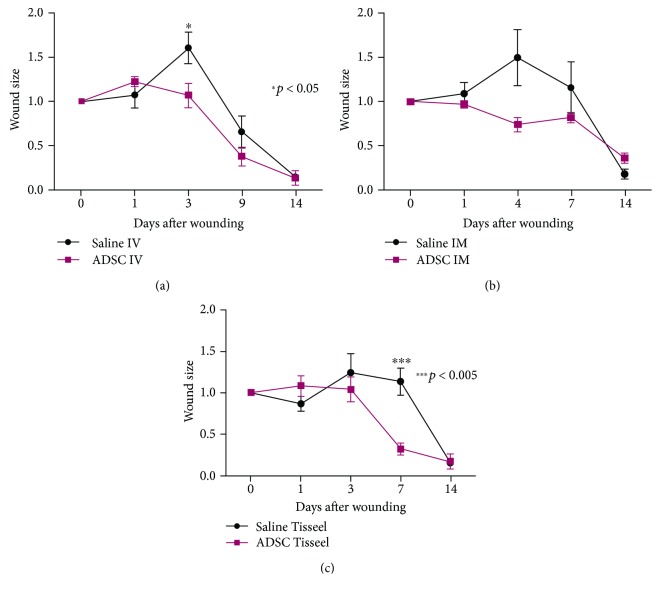
The size of the wound was monitored every day until day 14. Results are expressed as means of wound size. (a) Wound size after intravenous injection. Intravenous injection of ASCs was seen to accelerate wound healing on day 3 with significance (wound size of the ASC group 1.07 ± 0.27 vs. the control group 1.61 ± 0.40; *p* = 0.046). (b) Wound size after intramuscular injection. Intramuscular injection of ASCs accelerated wound healing mostly on day 4 with significance (wound size of the ASC group 0.74 ± 0.16 vs. control group 1.50 ± 0.64; *p* = 0.05). (c) Wound size after topical application. The topical application of ASCs accelerated the wound closure compared to the control group on day 3 (wound size of the ASC group 1.04 ± 0.28 vs. the control group 1.24 ± 0.46; *p* = 0.476) and day 7 (wound size of the ASC group 0.32 ± 0.05 vs. the control group 1.13 ± 0.36; *p* = 0.05) (*p* = 0.001).

Intravenous injection of ASCs (Figure 3(a)) was seen to accelerate wound healing on day 3 (wound size of the ASC group 1.07 ± 0.27 vs. the control group 1.61 ± 0.40; *p* = 0.046), and intramuscular injection of ASCs (Figure 3(b)) also accelerated wound healing on day 4 (wound size of the ASC group 0.74 ± 0.16 vs. the control group 1.50 ± 0.64; *p* = 0.05), while topical application of ASCs also accelerated wound healing on day 3 (wound size of the ASC group 1.04 ± 0.28 vs. the control group 1.24 ± 0.46; *p* = 0.476) and day 7 (wound size of the ASC group 0.32 ± 0.05 vs. the control group 1.13 ± 0.36; *p* = 0.05) (*p* = 0.001) (Figure 3(c)). Overall, acceleration of wound healing in response to ASCs was most evident on postoperative days 3-4. On day 14, the wounds of all the groups receiving ASCs were completely healed, whereas in the controls healing was incomplete.

### 3.3. Histological Analysis

**Figure 4:**
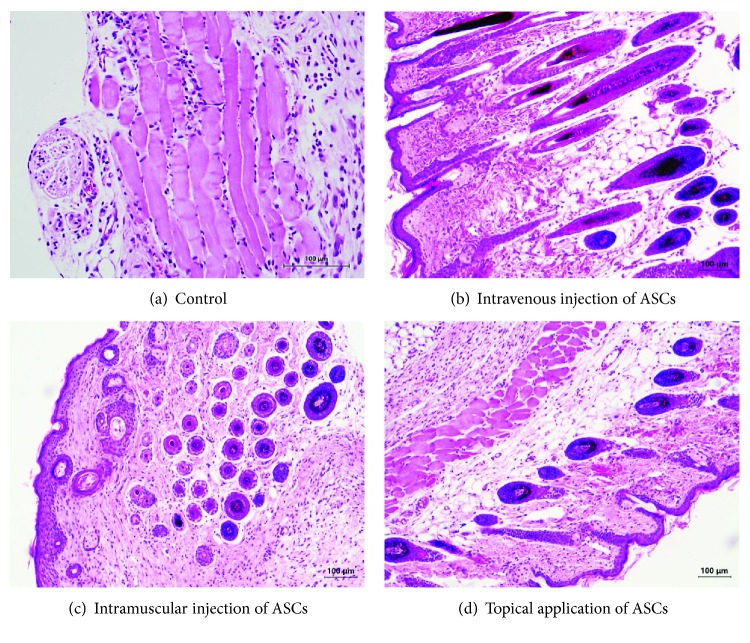
Histological observations showing that tissue regeneration was much more extensive in the ASC groups than in the control groups. (a) In the control group, epithelium is not formed. The dermis layer has less vascularization, and inflammatory cell infiltration still occurred. (b, c, and d) The biopsies from the ASC-treated mice presented a good stratified and differentiated epithelial layer. The epidermis and dermis layers were well formed in layers with adequate vascularity and less inflammation.

Histological analysis revealed that tissue regeneration was accelerated in the ASC-treated mice. In the controls (Figure 4(a)), epithelium was not formed, the dermis appeared less vascularized, and inflammatory cell infiltration still occurred. All the biopsies from the ASC-treated mice presented a good stratified and differentiated epithelial layer. The epidermis and dermis layers were well formed with adequate vascularity and less inflammation (Figures 4(b)–4(d)).

### 3.4. Migration of ASCs into the Engraftment

In the intravenous injection group and the intramuscular injection group, GFP-labelled ASCs were examined using an IVIS imaging system to determine whether the administered ASCs were incorporated into the wound area. In the case of the topical application group, as ASCs were directly spread over the wound, this examination was unnecessary.

**Figure 5:**
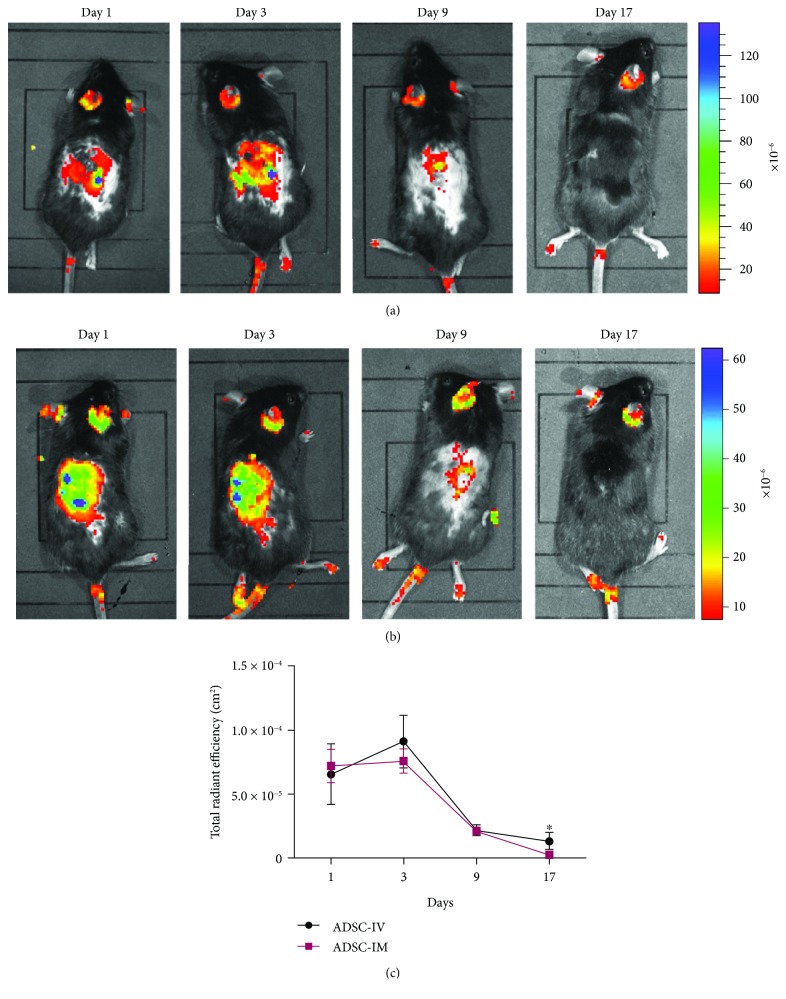
Detection of ASCs with an IVIS imaging system. (a) Intravenously injected ASCs gathered in the wound area until the wounds healed. (b) Intramuscularly injected ASCs remained in the wound area until the wounds healed. (c) GFP signals persisted while wound healing is underway and declined rapidly after postoperative day 9 (^∗^*p* < 0.05).

We detected large numbers of GFP-labelled ASCs in the wound areas of the ASC-treated mice (Figures 5(a) and 5(b)). However, the number of these cells declined rapidly after day 9 (Figure 5(c)). The mechanism underlying this reduction may be related to the progression of the wound healing process.

## 4. Discussion

Wound healing is a complex process involving inflammation, proliferation, and remodeling [11, 13, 17]. Impaired wound healing results from many conditions such as diabetes, chronic renal failure, autoimmune diseases, and radiation damage [18, 19]. The effects of compromised wound healing such as constant inflammation, decreased cell infiltration, and reduced levels of growth factors and cytokines, all lead to defective progression through the normal stages of wound healing. However, because many different factors can cause chronic wound healing, it is difficult to identify its primary cause.

Cell therapy is an attractive approach for the treatment of nonhealing wounds [1, 2]. Stem cells are undifferentiated cells with self-renewing properties that are able to differentiate into multiple cell types. Mesenchymal stem cells can be isolated from various sites, including bone marrow, adipose tissue, cord blood, and amniotic fluid. [5–7] They share many properties with mesenchymal stem cells from bone marrow and can differentiate into vascular endothelial cells, fibroblasts, osteoblasts, chondroblasts, and neurocytes [10, 20, 21]. ASCs are easier to isolate and are relatively abundant, which make them a potential resource for wound repair and regeneration [5, 11]. Previous studies have demonstrated that ADCs survive better than mature cells under hypoxia and mechanical stress, and in low nutrients. In addition, they have the ability to migrate into sites of inflammation to stimulate the proliferation and differentiation of progenitor cells, to promote the recovery of injured cells by secreting growth factors and cytokines, and to have immunomodulatory and anti-inflammatory effects [2, 7, 22, 23]. All these characteristics promote wound healing.

We used a mouse model with an excisional wound to examine the effects of ASCs on wound healing. We excised quite a large wound area (1 × 1 cm) and sutured the margin to a deep muscle layer to avoid wound contraction and to allow wound healing by granulation and epithelialization.

The method of cell administration can affect the distribution of injected cells in the body [18, 19, 24, 25]. We aimed to compare what happened to the injected cells and how they affected wound healing, when they were injected systemically, injected locally, and administered topically. In a previous study using rodents, intravenously injected stem cells were found throughout the body, predominantly in the lungs and liver, but also in the spleen and other organs. In addition, stem cells can migrate to sites of inflammation, probably through paracrine mechanisms [26–28]. Local intramuscular injection in the injured area might be helpful and the wound only requires the precise amount of cells needed for the specific purpose. Furthermore, we applied the ASCs mixed with fibrin sealant topically directly over the wound as a structural support. The fibrinogen and fibronectin contained in the fibrin sealant can also stimulate the secretion of the extracellular matrix and the regeneration of host tissue. Topical application over the wound also shortens treatment time and is more accurate.

In our experiments, ADCs significantly accelerated the wound healing process, with more rapid reepithelialization and increased granulation tissue formation from day 3 to day 7; this reduced the time required for complete wound healing. We also observed a rapid reduction in the number of GFP-labelled ADCs after postoperative day 9, which may be related to the progression of wound healing.

Our results also indicated that many intravenously injected ADCs migrated to the excised wound area, and intramuscularly injected ASCs remained at the site of injection, like the topically administered ASCs. This further supports the idea that intravenously injected stem cells migrate to sites that require them and have similar effects to locally administered ASCs.

## 5. Conclusion

The present study clearly demonstrates that administration of ADCs into mice with a full-thickness wound, either intravenously, intramuscularly, or topically, accelerates wound healing. Interestingly, in addition to increasing the healing rate, ADCs improved the remodeling of wounds, with improved closure. These effects of ADCs did not depend on the method of administration.

## Data Availability

The data used to support the findings of this study are included within the article. If more data are required after the publication of this article, the request will be considered by the corresponding author.
