# Corrigendum to “The Effect of Adipose-Derived Stem Cells on Wound Healing: Comparison of Methods of Application”

**DOI:** 10.1155/2020/2193130

**Published:** 2020-04-08

**Authors:** Hyeonwoo Kim, Mi Ri Hyun, Sang Wha Kim

**Affiliations:** Department of Plastic and Reconstructive Surgery, College of Medicine, Seoul National University, Seoul, Republic of Korea

In the article titled “The Effect of Adipose-Derived Stem Cells on Wound Healing: Comparison of Methods of Application” [1], the affiliation should be changed from “Department of Plastic and Reconstructive Surgery, Seoul National University Hospital, Seoul, Republic of Korea” to the correct affiliation “Department of Plastic and Reconstructive Surgery, College of Medicine, Seoul National University, Seoul, Republic of Korea.” The corrected affiliation is shown in the author information above.
